# Risk of first peritonitis episode in continuous ambulatory peritoneal dialysis and automated peritoneal dialysis: a population-based study

**DOI:** 10.1093/ckj/sfae118

**Published:** 2024-04-25

**Authors:** Franco Wing Tak Cheng, Marco Chau, Xue Li, Jiahao Liang, Ian Chi Kei Wong, Sydney Chi Wai Tang

**Affiliations:** Centre for Safe Medication Practice and Research, Department of Pharmacology and Pharmacy, Li Ka Shing Faculty of Medicine, The University of Hong Kong, Hong Kong SAR, China; Centre for Safe Medication Practice and Research, Department of Pharmacology and Pharmacy, Li Ka Shing Faculty of Medicine, The University of Hong Kong, Hong Kong SAR, China; Centre for Safe Medication Practice and Research, Department of Pharmacology and Pharmacy, Li Ka Shing Faculty of Medicine, The University of Hong Kong, Hong Kong SAR, China; Laboratory of Data Discovery for Health (D24H), Hong Kong Science and Technology Park, Sha Tin, Hong Kong SAR, China; Department of Medicine, School of Clinical Medicine, Li Ka Shing Faculty of Medicine, The University of Hong Kong, Hong Kong SAR, China; Centre for Safe Medication Practice and Research, Department of Pharmacology and Pharmacy, Li Ka Shing Faculty of Medicine, The University of Hong Kong, Hong Kong SAR, China; Centre for Safe Medication Practice and Research, Department of Pharmacology and Pharmacy, Li Ka Shing Faculty of Medicine, The University of Hong Kong, Hong Kong SAR, China; Laboratory of Data Discovery for Health (D24H), Hong Kong Science and Technology Park, Sha Tin, Hong Kong SAR, China; Aston Pharmacy School, Aston University, Birmingham, UK; Department of Medicine, School of Clinical Medicine, Li Ka Shing Faculty of Medicine, The University of Hong Kong, Hong Kong SAR, China

To the Editor,

In Hong Kong, a peritoneal dialysis (PD)-first policy has been adopted for all patients with kidney failure requiring dialysis unless medically contraindicated [1]. PD is generally well-tolerated with better quality of life, better preserved residual kidney function, increased hemodynamic stability and a lower rate of blood-borne infections than hemodialysis (HD) [2]. Nevertheless, peritonitis is one major PD complication that could undermine dialysis and reduce quality of life, and is a major cause of morbidity and mortality [3, 4]. In addition, severe or repeated peritonitis can result in peritoneal membrane failure, leading to technique failure and conversion to chronic HD [5].

The International Society for Peritonitis Dialysis has published several recommendations to minimize peritonitis, but the adoption rate varies [6]. A much-debated question is whether the use of automated PD (APD) could lower the incidence of peritonitis compared with continuous ambulatory PD (CAPD), on account of a lower frequency of manual exchanges which could theoretically reduce the risk of contamination and hence the incidence of peritonitis [7]. In particular, even though APD usually requires fewer manual exchanges, multiple line connections are required for each exchange, theoretically increasing the risk of contamination. However, data regarding the impact of PD modalities on the risk of peritonitis remain contradictory [8]. We therefore conducted this study to evaluate the risk of first peritonitis episode among different PD systems.

Our study was a population-based, observational, retrospective cohort study using electronic medical records in the Hong Kong Hospital Authority. Adult patients who newly started PD between 2007 and 2019 were included. The exposure was PD modality, classified as APD, Disc System (Andy Disc^®^ and Stay Safe Disc^®^, Fresenius), Stay Safe Balance^®^ (Fresenius) and UltraBag^®^ (Baxter Healthcare). The primary outcome of interest was peritonitis, defined by diagnostic codes using the International Classification of Diseases, Ninth Revision, Clinical Modification (014.0, 032.83, 095.2, 098.86, 567.0, 567.1, 567.2, 567.89, 567.9, 996.68), with secondary outcomes of interest including all-cause mortality, cardiovascular death, all-cause accident and emergency department (AED) attendance and technique failure. Patients were followed from the date of the first outpatient or discharge prescription containing PD fluids until the date of outcome occurrence, changes in PD modality, conversion to HD or having been transplanted, discontinuation of PD, 3 years from the first prescription of PD fluid, or the end of the study period, whichever came first. We applied multi-group inverse probability of treatment weighting Cox proportional-hazards models and Kaplan–Meier curve to evaluate the hazard ratios (HRs) and to illustrate the cumulative incidence of the outcomes over time, respectively. Subgroup analyses and sensitivity analyses were also performed. Detailed methodology is described in Supplementary data, 1.

A total of 14 693 patients with a prescription of PD fluid were identified. After excluding patients without a discharge or outpatient prescription of PD fluid, who initiated PD at age <18 years or with a PD regimen containing either icodextrin, Spike or Twin-Bag, a record of 11 021 patients was retained and analysed (Supplementary data, 2).

More than 68.6% of the included patients were prescribed the Ultrabag^®^ system, while 9.7%, 12.0% and 9.6% of patients used the APD, Disc System and Stay Safe Balance^®^ systems, respectively (Supplementary data, 3). The adoption of different PD modalities evolved over the study period. The proportion of patients using the UltraBag^®^ system gradually reduced from 71.9% in 2007 to 53.2% in 2019, while APD increased from 6.2% in 2013 to 18.7% in 2019. The use of the Disc System reduced gradually with the increase of use of the newer Stay Safe Balance^®^ system over the study period (Supplementary data, 4). Age, sex and proportion of different comorbidities were similar in each group after matching.

Compared with APD, the other three systems showed increased risks of peritonitis [Disc System: HR 1.88 (95% confidence inverval, CI 1.51–2.33); Stay Safe Balance^®^: HR 2.22 (95% CI 1.76–2.80); UltraBag^®^: HR 1.93 (95% CI 1.61–2.33)], but not all-cause mortality and technique failure. APD also showed a reduced risk of AED attendance compared with Disc System [HR 1.30 (95% CI 1.10–1.55)] and Ultrabag^®^ [HR 1.45 (95% CI 1.26–1.66)] systems, but not the Stay Safe Balance^®^ [HR 0.87 (95% CI 0.70–1.07)] system (Table 1, Fig. 1).

**Figure 1:**
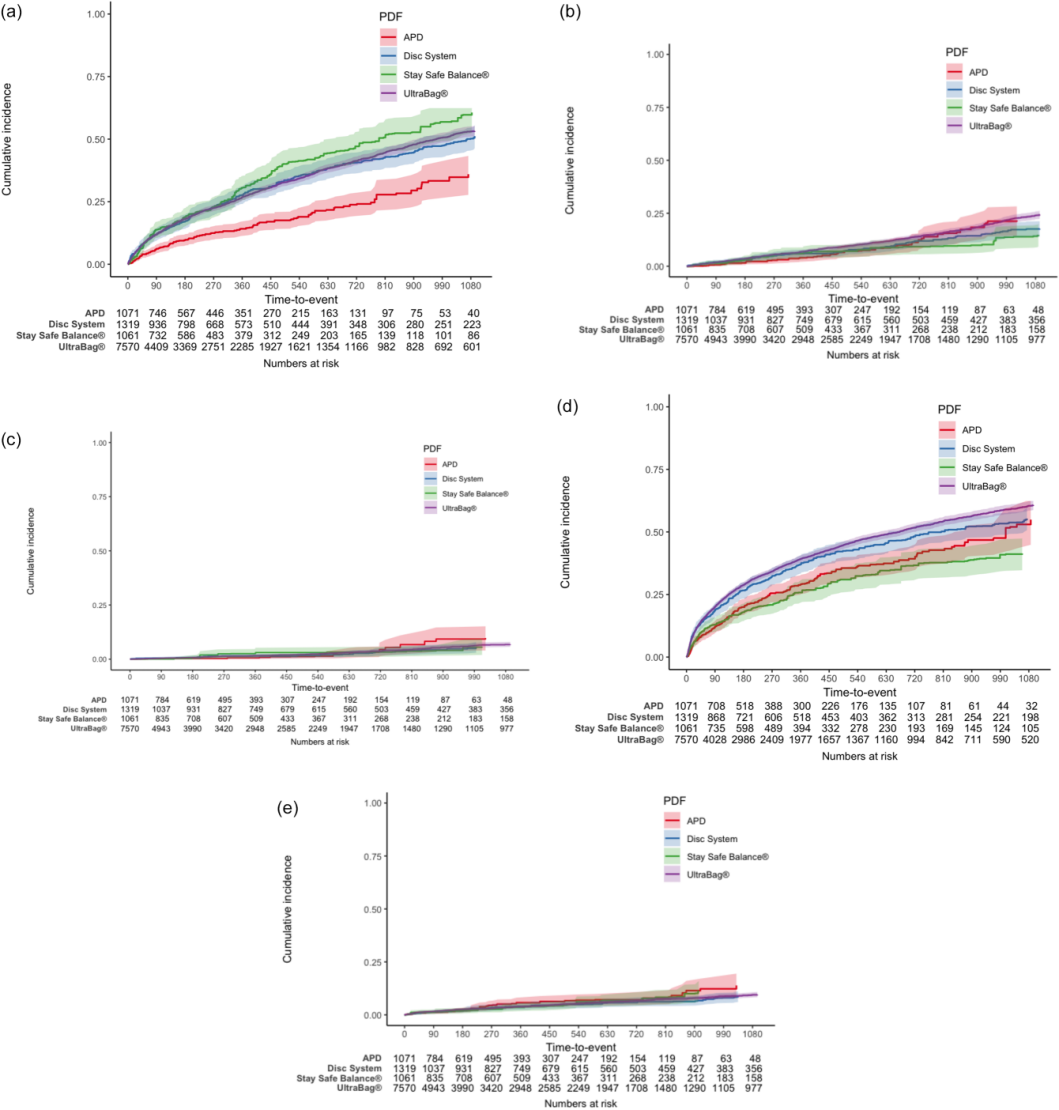
(**a**) Kaplan–Meier curve showing cumulative incidence of peritonitis in different groups. (**b**) Kaplan–Meier curve showing cumulative incidence of all-cause death in different groups. (**c**) Kaplan–Meier curve showing cumulative incidence of cardiovascular death in different groups. (**d**) Kaplan–Meier curve showing cumulative incidence of AED attendance in different groups. (**e**) Kaplan–Meier curve showing cumulative incidence of technique failure in different groups.

**Table 1:** Comparison of HR of peritonitis, all-cause mortality, cardiovascular mortality, AED attendance and technique failure among patients with APD and other CAPD modalities.

	*n*/*N*	Hazard ratio	95% CI	*P*-value
Peritonitis				
APD	142/1071	Ref		
Disc System	420/1319	1.88	(1.51–2.33)	<.001
Stay Safe Balance^®^	334/1061	2.22	(1.76–2.80)	<.001
UltraBag^®^	1877/7570	1.93	(1.61–2.33)	<.001
All-cause mortality				
APD	58/1071	Ref		
Disc System	123/1319	1.01	(0.70–1.46)	>.9
Stay Safe Balance^®^	69/1061	0.91	(0.57–1.45)	.700
UltraBag^®^	654/7570	1.35	(1.00–1.84)	.053
Cardiovascular death				
APD	18/1071	Ref		
Disc System	34/1319	0.83	(0.42–1.66)	.6
Stay Safe Balance^®^	25/1061	1.01	(0.45–2.27)	>.9
UltraBag^®^	161/7570	1.01	(0.59–1.73)	>.9
AED attendance				
APD	265/1071	Ref		
Disc System	496/1319	1.30	(1.10–1.55)	.003
Stay Safe Balance^®^	268/1061	0.87	(0.70–1.07)	.2
UltraBag^®^	2513/7570	1.45	(1.26–1.66)	<.001
Technique failure				
APD	55/1071	Ref		
Disc System	73/1319	0.72	(0.47–1.09)	.12
Stay Safe Balance^®^	53/1061	0.82	(0.51–1.32)	.4
UltraBag^®^	346/7570	0.80	(0.58–1.10)	.2

The subgroup analyses and sensitivity analyses are largely consistent with the main analysis, except for an increased risk of all-cause mortality [HR 1.72 (95% CI 1.10–2.68)] and cardiovascular death [HR 3.05 (95% CI 1.08–8.59)] observed in male patients using the UltraBag^®^ system. A reduction in risk of cardiovascular death in female patients [Disc System: HR 0.28 (95% CI 0.11–0.68); Stay Safe Balance^®^: HR 0.45 (95% CI 0.11–1.83); UltraBag^®^: HR 0.46 (95% CI 0.23–0.92)] was also observed (Supplementary data, 5 and 6).

The current study found that patients undergoing APD have a lower risk of peritonitis compared with the other three CAPD systems. We also observed a lower risk of AED attendance using APD when compared with UltraBag^®^ and Disc System. However, no difference in all-cause mortality, cardiovascular death and technique failure was observed among different PD systems. Compared with APD, the risks of all-cause mortality and cardiovascular death are higher in male patients using the UltraBag^®^ system.

The reduction of risk of peritonitis can be explained by the fewer manual exchanges necessary for APD than CAPD. Our finding is consistent with previous studies from Taiwan and Mexico, both showing a reduced risk of peritonitis in APD compared with CAPD [9, 10]. On the other hand, a cohort study in Brazil found no difference in time until the first peritonitis episode between APD and CAPD modalities [11].

Compared with HD, PD is associated with increased rates of hospital admission and in-hospital morbidities, mainly due to peritonitis and cardiovascular complications [12]. Hence, lowering the incidence of peritonitis may also decrease the frequency of AED attendance among patients using APD, as illustrated in our study.

Studies in the USA and Brazil have found better survival in patients undergoing APD [11, 13]. However, our study could not find a clear association between PD modalities and mortality. The relatively short follow-up period in our study may have limited the potential long-term survival benefits of APD. Another contributing factor could arise from the PD-first policy in Hong Kong, which selects younger and fitter patients with better preserved residual kidney function and hence a lower mortality rate at baseline.

In subgroup analyses, we found that the mortality was significantly higher in male patients using the UltraBag^®^ system compared with APD, where the association was not found in female patients. The risk of cardiovascular death was also significantly higher in male patients using the UltraBag^®^ system compared with APD, while the opposite was found in female patients. These unexpected results demonstrate the possibility that sex has an impact on the relationship between PD modalities and mortality. Further research investigating the impact of sex in the relationship between PD modalities and mortality is necessary.

This study stands out as being the largest investigation into the relationship between different PD modalities and common PD outcomes, covering from 2007 to 2019, and encompasses over 10 000 patients. There are several limitations in the study. First, although propensity score weighting was performed to reduce confounding factors, residual confounders (e.g. improving education and patient technique during manual exchanges) may still exist. Secondly, the severity of peritonitis could not be assessed and analysed with the use of electronic data. Thirdly, a recent study showed that the number of daily manual exchanges in CAPD was associated with the risk of peritonitis [14] but these data could not be incorporated into the current analysis. A different study design is warranted to further explore the effect of the incremental approach in CAPD compared with APD. Fourthly, patients could switch to other PD modalities during the study period, but they would be censored once they changed the PD modalities. This limited the study's power to detect differences in outcomes. Lastly, there is a significant discrepancy in the number of patients in different groups since the cost of APD cyclers is not reimbursed in Hong Kong. Further studies are required to illustrate the cost-effectiveness of APD.

In conclusion, the current study found that among incident PD patients, APD was associated with a lower risk of first peritonitis compared with other CAPD modalities. Further studies are warranted to elucidate the association between PD modalities and the risk of mortality.

## Supplementary Material

sfae118_Supplemental_File
