# Supplementary material for: Risk of first peritonitis episode in continuous ambulatory peritoneal dialysis and automated peritoneal dialysis: a population-based study
Source: Clin Kidney J. 2024 Apr 25;17(5):sfae118. doi: 10.1093/ckj/sfae118 (PMC11089411; doi:10.1093/ckj/sfae118)
Supplement: sfae118_Supplemental_File [file sfae118_supplemental_file.docx]

Table of Contents

**Supplementary Material 1. Methodology …………………………………………………………… 2**

**Supplementary Material 2. Baseline characteristics of patients started APD or other CAPD modalities before and after propensity score weighting ……………………………………………. 5**

**Supplementary Material 3. Inclusion and exclusion criteria for cohort analysis of patients who used automated peritoneal dialysis (APD) and other continuous ambulatory peritoneal dialysis modalities from 1 January 2007 to 31 December 2019 ……………………………………………... 7**

**Supplementary Material 4. Uptake of patients during the study cohort, classified by APD or other CAPD modalities …………………………………………………………………………………….... 8**

**Supplementary Material 5. Hazard ratio of peritonitis and mortality among patients with APD
and other CAPD modalities ………………...……………………………………………………...… 9**

**Supplementary Material 6. Hazard ratio of peritonitis, all-cause mortality, cardiovascular
mortality, AED attendance and technique failure among patients with APD and other
CAPD modalities using different analyses ………………………………………………………...... 10**

**Supplementary Material 1. Methodology**

*Study design*

This study was a population-based, observational, retrospective cohort study. We aimed to investigate the risk of peritonitis in patients receiving different PD modalities. From the electronic medical records, PD modalities were determined by the type of PD fluid prescribed.

*Data source*

Electronic medical records were retrieved from the electronic clinical database in the Hong Kong Hospital Authority (HA). HA is a major publicly funded healthcare provider in Hong Kong, managing all publicly funded hospitals and clinics. It covers more than 90% of the KRT patients in Hong Kong. The records included patient demographics, date of dialysis access creation, complications, hospitalization records, key performance parameters such as adequacy, ESA usage and other pertinent laboratory test results, other clinical diagnoses, and drug dispensing records (including PD fluid). A unique patient reference number was generated for each patient to link all data sources and facilitate the procedure of data retrieval to ensure that the records were anonymized.

*Study population*

Adult patients who newly initiated PD and were followed up in hospitals under HA between 1 January 2007 and 31 December 2019 were included in this study. Patients using PD in 2006 but stopped for at least 1 year and subsequently resumed within the study period were also included. Patients who initiated PD at age <18, initiated a PD regimen containing icodextrin or the obsolete Spike or Twin-bag^TM^ PD modalities were excluded.

*Exposure*

The exposure was PD modality, which is determined by the type of PD fluid prescribed in the drug dispensing records. Four PD modalities were classified, namely APD, Disc System (Andy Disc® and Stay Safe Disc®, Fresenius), Stay Safe Balance® (Fresenius) and UltraBag® (Baxter Healthcare). The follow-up period was from the date of the first outpatient or discharge prescription containing PD fluids until the date of outcome occurrence (first peritonitis or death), changes in PD modality, conversion to HD or having been transplanted, discontinuation of PD, 3 years from the first prescription of PD fluid, or the end of study period (31 December 2019), whichever came first.

*Outcomes*

The primary outcome of interest was peritonitis, defined according to the International Classification of Disease, Ninth Revision, Clinical Modification (ICD-9-CM: 014.0, 032.83, 095.2, 098.86, 567.0, 567.1, 567.2, 567.89, 567.9, 996.68). The event date was determined as the date of first hospital admission with the diagnosis of peritonitis. The secondary outcomes of interest included all-cause mortality, cardiovascular death, all-cause accident and emergency department (AED) attendance and technique failure. Technique failure was defined as a switch from PD to HD for at least 30 days.

*Statistical analysis*

Since the demographics and comorbidities of patients with different PD modalities differed, multi-group Inverse Probability of Treatment Weighting (IPTW) was adopted to ascertain the balance of patient baseline characteristics across groups (Table 1). Weighted variables included demographics such as age and sex; Charlson comorbidity index (CCI) and non-CCI clinical conditions including asthma, stroke or systemic embolism, hypertension and severe constipation. The weighting was conducted using the function "weightit" in the R-package "WeightIt". Weighted results were two-sided trimmed by 1% of extreme values. We applied weighted Cox proportional-hazards models to evaluate the hazard ratios and their 95% confidence intervals (CI) using APD as the reference group. Kaplan–Meier estimates were prepared to illustrate the cumulative incidence of first peritonitis and death over time. Separate analyses were conducted for the primary and secondary outcomes. Subgroup analyses were also conducted for different entry age (<65 and ≥65), sex and diabetes status. We also conducted three sensitivity analyses to ensure the robustness of the study, namely (i) including the start year of PD as a covariate; (ii) including the start year of PD, sex and age categories as covariates; (iii) including the start year of PD, and all baseline characteristics as covariates and (iv) using entropy balancing instead of propensity score as weighting estimators.^14^ A two-sided significance level of 5% was used in all statistical analyses. All statistical tests were two-sided and p-values of less than 0.05 were considered significant. Statistical analysis was considered using R version 4.0.5.

*Ethical approval*

Ethical approval for this study was granted by the Institutional Review Board of the University of HK/HA HK West Cluster (UW 20-110).

**Supplementary Material 2. Baseline characteristics of patients started APD or other CAPD modalities before and after propensity score weighting**

|  | **Before weighting** | | | | | **After weighting** | | | | |
| --- | --- | --- | --- | --- | --- | --- | --- | --- | --- | --- |
|  | APD | Disc System | Stay Safe Balance® | UltraBag® | Max SMD | APD | Disc System | Stay Safe Balance® | UltraBag® | Max SMD |
| N | 1071 | 1319 | 1061 | 7570 |  | 734.97 | 904.79 | 741.57 | 5199.61 |  |
| Median follow-up duration, months (IQR) | 9.4 (4.1-18.9) | 20.5 (6.9-43.7) | 12.6 (5.3-26) | 9.5 (2.4-25.4) |  |  |  |  |  |  |
| Age (SD) | 58.65 (15.09) | 63.66  (12.19) | 59.67  (12.32) | 60.71  (12.11) | 0.207 | 60.76  (12.31) | 60.26 (12.64) | 60.91  (12.17) | 60.71 (12.11) | 0.027 |
| Sex, male (%) | 638  (59.6) | 697  (52.8) | 588  (55.4) | 4833  (63.8) | 0.126 | 471.6  (64.2) | 568.6 (62.8) | 490.6 (66.2) | 3319.6 (63.8) | 0.036 |
| AIDS (%) | 2 (0.2) | 1 (0.1) | 1 (0.1) | 8 (0.1) | 0.016 | 1.1 (0.2) | 0.7 (0.1) | 0.4 (0.1) | 5.5 (0.1) | 0.016 |
| Asthma (%) | 22 (2.1) | 25 (1.9) | 21 (2.0) | 111 (1.5) | 0.023 | 11.9 (1.6) | 12.3(1.4) | 9.9 (1.3) | 76.2 (1.5) | 0.013 |
| Cancer (%) | 51 (4.8) | 102 (7.7) | 56 (5.3) | 408 (5.4) | 0.062 | 41.5 (5.6) | 51.3 (5.7) | 34.4(4.6) | 280.2 (5.4) | 0.025 |
| Metastatic (%) | 3 (0.3) | 5 (0.4) | 3 (0.3) | 26 (0.3) | 0.010 | 3.5 (0.5) | 3.5 (0.4) | 1.4 (0.2) | 17.9 (0.3) | 0.027 |
| Cerebrovascular disease (%) | 137  (12.8) | 197  (14.9) | 110  (10.4) | 1054  (13.9) | 0.074 | 109.3  (14.9) | 127.3 (14.1) | 95.0  (12.8) | 724.0  (13.9) | 0.030 |
| Stroke (%) | 85 (7.9) | 122 (9.6) | 65 (6.1) | 706 (9.3) | 0.068 | 72.5 (9.9) | 82.8 (9.2) | 66.6 (9.0) | 484.2 (9.3) | 0.016 |
| COPD (%) | 48 (4.5) | 71 (5.4) | 51 (4.8) | 304 (4.0) | 0.035 | 30.2 (4.1) | 33.9 (3.7) | 30.2 (4.1) | 208.8 (4.0) | 0.010 |
| CHF (%) | 147 (13.7) | 255 (19.3) | 98 (9.2) | 1431 (18.9) | 0.169 | 142.6 (19.4) | 169.9 (18.8) | 144.8 (19.5) | 982.9 (18.9) | 0.012 |
| Dementia (%) | 3 (0.3) | 8 (0.6) | 1 (0.1) | 27 (0.4) | 0.047 | 2.9 (0.4) | 3.3 (0.4) | 0.7 (0.1) | 18.5 (0.4) | 0.031 |
| Diabetes mellitus |  |  |  |  |  |  |  |  |  |  |
| Without complications (%) | 422 (39.4) | 414 (31.4) | 200 (18.9) | 3626 (47.9) | 0.348 | 367.8 (50.0) | 467.6 (51.7) | 358.3 (48.3) | 2490.6 (47.9) | 0.044 |
| With complications (%) | 382 (35.7) | 367 (27.8) | 172 (16.2) | 3646 (48.2) | 0.387 | 328.7 (44.7) | 418.8 (46.3) | 305.3 (41.2) | 2504.3 (48.2) | 0.076 |
| Hypertension (%) | 732 (68.3) | 984 (74.6) | 693 (65.3) | 5669 (74.9) | 0.128 | 549.9 (74.8) | 682.5 (75.4) | 553.4 (74.6) | 3893.9 (74.9) | 0.010 |
| Liver disease |  |  |  |  |  |  |  |  |  |  |
| Mild (%) | 7 (0.7) | 18 (1.4) | 11 (1.0) | 98 (1.3) | 0.040 | 9.1 (1.2) | 11.0 (1.2) | 13.8 (1.9) | 67.3 (1.3) | 0.027 |
| Moderate or severe (%) | 3 (0.3) | 5 (0.4) | 3 (0.3) | 43 (0.6) | 0.025 | 5.6 (0.8) | 3.9 (0.4) | 6.1 (0.8) | 29.5 (0.6) | 0.029 |
| MI (%) | 80 (7.5) | 95 (7.2) | 59 (5.6) | 690 (9.1) | 0.070 | 66.4 (9.0) | 77.1 (8.5) | 66.4 (9.0) | 473.9 (9.1) | 0.011 |
| Paralysis (%) | 25 (2.3) | 36 (2.7) | 14 (1.3) | 161 (2.1) | 0.053 | 15.9 (2.2) | 21.8 (2.4) | 16.4 (2.2) | 110.6 (2.1) | 0.010 |
| PVD (%) | 26 (2.4) | 34 (2.6) | 36 (3.4) | 283 (3.7) | 0.046 | 24.8 (3.4) | 29.2 (3.2) | 24.2 (3.3) | 194.4 (3.7) | 0.015 |
| Rheumatic Diseases (%) | 5 (0.5) | 7 (0.5) | 1 (0.1) | 31 (0.4) | 0.041 | 3.4 (0.5) | 3.2 (0.4) | 1.8 (0.2) | 21.3 (0.4) | 0.019 |
| Severe Constipation (%) | 76 (7.1) | 116 (8.8) | 91 (8.6) | 691 (9.1) | 0.039 | 68.1 (9.3) | 76.6 (8.5) | 70.7 (9.5) | 474.6 (9.1) | 0.019 |
| Ulcers (%) | 78 (7.3) | 127 (9.6) | 85 (8.0) | 728 (9.6) | 0.052 | 67.1 (9.1) | 83.5 (9.2) | 79.4 (10.7) | 500.0 (9.6) | 0.029 |
| CCI (SD) | 3.50 (1.55) | 3.48 (1.61) | 2.98 (1.32) | 3.88 (1.55) | 0.307 | 3.88 (1.65) | 3.85 (1.59) | 3.79 (1.52) | 3.88 (1.55) | 0.031 |

APD, Automated peritoneal dialysis; CCI, Charlson comorbidity index; CHF, congestive heart failure; COPD, chronic obstructive pulmonary disease; IQR, interquartile range; MI, myocardial infarction; PVD, peripheral vascular disease; SD, standard deviation; SMD, standardized mean difference.

**Supplementary Material 3. Inclusion and exclusion criteria for cohort analysis of patients who used automated peritoneal dialysis (APD) and other continuous ambulatory peritoneal dialysis modalities from 1 January 2007 to 31 December 2019**

Patients who initiated PD from 1 January 2007 to 31 December 2019 or who used in 2006 but stopped for ≥365 days before resume (n = 14693)

Patients who used PD but without discharge / outpatient records (n = 1723)

Patients who initiated PD from 1 January 2007 to 31 December 2019 at discharge or out-patient or who used in 2006 but stopped for ≥365 days before resume (n = 12970)

Patients who were age <18 when start PD (n = 69)
Patients who started with icodextrin regimen (n = 829)
Patients who started with Spike regimen (n = 624)
Patients who started with Twin-Bag regimen (n = 140)
Patients who started with unclassified PD regimen (n = 76)

Adults who initiated PD from 1 January 2007 to 31 December 2019 at discharge or out-patient or who used in 2006 but stopped for ≥365 days before resume (n = 11,021)

- APD (n = 1,071)
- Stay Safe (n = 1,319)
- Stay Safe Balance (n = 1,061)
- UltraBag (n = 7,570)

APD, automated peritoneal dialysis; PD, peritoneal dialysis.

**Supplementary Material 4. Uptake of patients during the study cohort, classified by APD or other CAPD modalities**

APD, automated peritoneal dialysis.

**Supplementary Material 5. Hazard ratio of peritonitis and mortality among patients with APD and other CAPD modalities**

|  | **Peritonitis** | | **All-cause Mortality** | | **Cardiovascular death** | | **AED attendance** | | **Technique failure** | |
| --- | --- | --- | --- | --- | --- | --- | --- | --- | --- | --- |
|  | HR^a^ | 95% CI^a^ | HR^a^ | 95% CI^a^ | HR^a^ | 95% CI^a^ | HR^a^ | 95% CI^a^ | HR^a^ | 95% CI^a^ |
| Male | | | | | | | | | | |
| Disc System | 2.17 | (1.61 - 2.93) | 1.41 | (0.84 - 2.38) | 2.89 | (0.89 - 9.34) | 1.52 | (1.20 - 1.92) | 0.69 | (0.41 - 1.18) |
| Stay Safe Balance® | 2.28 | (1.66 - 3.13) | 1.17 | (0.63 - 2.17) | 3.01 | (0.84 - 10.8) | 0.91 | (0.68 - 1.21) | 0.79 | (0.44 - 1.43) |
| UltraBag® | 2.05 | (1.59 - 2.63) | 1.72 | (1.10 - 2.68) | 3.05 | (1.08 - 8.59) | 1.56 | (1.29 - 1.88) | 0.76 | (0.51 - 1.14) |
| Female | | | | | | | | | | |
| Disc System | 1.58 | (1.15 - 2.18) | 0.66 | (0.38 - 1.14) | 0.28 | (0.11 - 0.68) | 1.04 | (0.80 - 1.35) | 0.70 | (0.35 - 1.42) |
| Stay Safe Balance® | 2.28 | (1.58 - 3.28) | 0.52 | (0.22 - 1.20) | 0.45 | (0.11 - 1.83) | 0.80 | (0.57 - 1.13) | 0.88 | (0.39 - 1.97) |
| UltraBag® | 1.88 | (1.41 - 2.50) | 0.99 | (0.63 - 1.56) | 0.46 | (0.23 - 0.92) | 1.30 | (1.06 - 1.61) | 0.93 | (0.51 - 1.70) |
| Age ≥ 65 | | | | | | | | | | |
| Disc System | 2.07 | (1.54 - 2.78) | 0.87 | (0.56 - 1.35) | 0.41 | (0.18 - 0.95) | 1.14 | (0.90 - 1.44) | 1.10 | (0.56 - 2.16) |
| Stay Safe Balance® | 2.59 | (1.87 - 3.60) | 0.88 | (0.49 - 1.58) | 0.50 | (0.15 - 1.67) | 0.89 | (0.65 - 1.22) | 1.19 | (0.53 - 2.70) |
| UltraBag® | 1.94 | (1.49 - 2.52) | 1.31 | (0.90 - 1.90) | 0.78 | (0.42 - 1.45) | 1.40 | (1.15 - 1.70) | 0.87 | (0.48 - 1.56) |
| Age < 65 | | | | | | | | | | |
| Disc System | 1.85 | (1.34 - 2.57) | 1.24 | (0.63 - 2.46) | 2.04 | (0.54 - 7.67) | 1.43 | (1.11 - 1.83) | 0.58 | (0.33 - 1.03) |
| Stay Safe Balance® | 1.85 | (1.27 - 2.70) | 0.87 | (0.36 - 2.08) | 2.62 | (0.61 - 11.3) | 0.79 | (0.57 - 1.11) | 0.58 | (0.32 - 1.07) |
| UltraBag® | 2.01 | (1.54 - 2.63) | 1.45 | (0.81 - 2.61) | 1.81 | (0.55 - 5.90) | 1.49 | (1.22 - 1.83) | 0.77 | (0.51 - 1.15) |
| Diabetes | | | | | | | | | | |
| Disc System | 1.82 | (1.36 - 2.45) | 1.05 | (0.66 - 1.65) | 0.93 | (0.40 - 2.14) | 1.26 | (1.00 - 1.57) | 0.58 | (0.32 - 1.04) |
| Stay Safe Balance® | 2.21 | (1.60 - 3.05) | 0.91 | (0.50 - 1.65) | 1.12 | (0.41 - 3.03) | 0.75 | (0.55 - 1.02) | 0.74 | (0.37 - 1.47) |
| UltraBag® | 1.93 | (1.52 - 2.46) | 1.44 | (1.00 - 2.08) | 1.23 | (0.64 - 2.37) | 1.37 | (1.15 - 1.63) | 0.76 | (0.49 - 1.16) |
| Non-diabetes | | | | | | | | | | |
| Disc System | 1.87 | (1.38 - 2.53) | 0.77 | (0.41 - 1.46) | 0.42 | (0.12 - 1.49) | 1.43 | (1.11 - 1.84) | 0.88 | (0.52 - 1.49) |
| Stay Safe Balance® | 2.23 | (1.65 - 3.01) | 0.80 | (0.41 - 1.58) | 0.59 | (0.19 - 1.84) | 1.25 | (0.97 - 1.63) | 0.93 | (0.55 - 1.56) |
| UltraBag® | 1.88 | (1.42 - 2.50) | 1.13 | (0.64 - 2.00) | 0.48 | (0.18 - 1.25) | 1.69 | (1.35 - 2.12) | 0.85 | (0.54 - 1.32) |

AED, accident and emergency department; CI, confidence interval; HR, hazard ratio.

* versus automated peritoneal dialysis

**Supplementary Material 6. Hazard ratio of peritonitis, all-cause mortality, cardiovascular mortality, AED attendance and technique failure among patients with APD and other CAPD modalities using different analyses**

|  | APD | Disc System | Stay Safe Balance® | UltraBag® |
| --- | --- | --- | --- | --- |
| *Unadjusted HR* | | | | |
| Peritonitis | Ref | 1.88 (1.51-2.33) | 2.22 (1.76-2.80) | 1.93 (1.61-2.33) |
| All-cause mortality | Ref | 1.01 (0.70-1.46) | 0.91 (0.57-1.45) | 1.35 (1.00-1.84) |
| Cardiovascular death | Ref | 0.83 (0.42-1.66) | 1.01 (0.45-2.27) | 1.01 (0.59-1.73) |
| AED attendance | Ref | 1.30 (1.10-1.55) | 0.87 (0.70-1.07) | 1.45 (1.26-1.66) |
| Technique failure | Ref | 0.72 (0.47-1.09) | 0.82 (0.51-1.32) | 0.80 (0.58-1.10) |
| *Model 1: Adjusted for year of PD initiation* | | | | |
| Peritonitis | Ref | 1.78 (1.42-2.24) | 2.20 (1.74-2.77) | 1.85 (1.52-2.23) |
| All-cause mortality | Ref | 0.98 (0.67-1.45) | 0.90 (0.56-1.44) | 1.34 (0.97-1.84) |
| Cardiovascular death | Ref | 0.93 (0.44-1.96) | 1.06 (0.46-2.42) | 1.06 (0.60-1.88) |
| AED attendance | Ref | 1.20 (1.00-1.44) | 0.85 (0.69-1.06) | 1.38 (1.19-1.59) |
| Technique failure | Ref | 0.75 (0.48-1.17) | 0.85 (0.53-1.37) | 0.82 (0.58-1.15) |
| *Model 2: Model 1 + Age + Sex* | | | | |
| Peritonitis | Ref | 1.76 (1.40-2.21) | 2.25 (1.78-2.84) | 1.82 (1.50-2.21) |
| All-cause mortality | Ref | 0.98 (0.67-1.45) | 0.91 (0.57-1.47) | 1.34 (0.97-1.84) |
| Cardiovascular death | Ref | 0.93 (0.44-1.94) | 1.08 (0.47-2.44) | 1.07 (0.61-1.88) |
| AED attendance | Ref | 1.19 (0.99-1.43) | 0.85 (0.69-1.06) | 1.37 (1.19-1.59) |
| Technique failure | Ref | 0.72 (0.46-1.12) | 0.83 (0.52-1.34) | 0.80 (0.57-1.13) |
| *Model 3: Model 1 + all baseline characteristics* | | | | |
| Peritonitis | Ref | 1.76 (1.40-2.22) | 2.26 (1.78-2.86) | 1.84 (1.52-2.24) |
| All-cause mortality | Ref | 1.02 (0.68-1.51) | 0.95 (0.58-1.56) | 1.45 (1.05-2.01) |
| Cardiovascular death | Ref | 0.94 (0.44-2.02) | 1.17 (0.51-2.72) | 1.20 (0.68-2.12) |
| AED attendance | Ref | 1.19 (0.99-1.43) | 0.86 (0.69-1.07) | 1.40 (1.21-1.61) |
| Technique failure | Ref | 0.71 (0.46-1.10) | 0.82 (0.51-1.32) | 0.80 (0.57-1.11) |
| *Model 4: Unadjusted HR using entropy balancing (instead of PS)* | | | | |
| Peritonitis | Ref | 1.89 (1.52-2.35) | 2.23 (1.77-2.82) | 1.95 (1.62-2.34) |
| All-cause mortality | Ref | 1.01 (0.69-1.46) | 0.90 (0.56-1.45) | 1.34 (0.98-1.82) |
| Cardiovascular death | Ref | 0.79 (0.39-1.59) | 0.94 (0.42-2.11) | 0.97 (0.56-1.67) |
| AED attendance | Ref | 1.32 (1.11-1.57) | 0.87 (0.70-1.08) | 1.46 (1.27-1.68) |
| Technique failure | Ref | 0.72 (0.47-1.08) | 0.82 (0.51-1.31) | 0.79 (0.58-1.10) |

AED, accident and emergency department; APD, automated peritoneal dialysis; HR, hazard ration; PD, peritoneal dialysis; PS, propensity score.
